# The Fis Protein Has a Stimulating Role in Initiation of Replication in *Escherichia coli In Vivo*

**DOI:** 10.1371/journal.pone.0083562

**Published:** 2013-12-16

**Authors:** Ingvild Flåtten, Kirsten Skarstad

**Affiliations:** Department of Cell Biology, Institute for Cancer Research, The Norwegian Radiumhospital, Oslo University Hospital, Oslo, Norway; University of Massachusetts, United States of America

## Abstract

The Fis protein is a nucleoid associated protein that has previously been reported to act negatively in initiation of replication in *Escherichia coli*. In this work we have examined the influence of this protein on the initiation of replication under different growth conditions using flow cytometry. The Fis protein was found to be increasingly important with increasing growth rate. During multi-fork replication severe under-initiation occurred in cells lacking the Fis protein; the cells initiated at an elevated mass, had fewer origins per cell and the origins were not initiated in synchrony. These results suggest a positive role for the Fis protein in the initiation of replication.

## Introduction

The duplication of the hereditary material before the cell divides is a fundamental process in all living cells. In *E.coli* cells the initiation of replication is a highly regulated process with many different players involved (see [1] for review). The main player in this process is the initiator protein DnaA, which binds to specific DnaA boxes within *oriC* [2]–[5]. This leads to formation of a DnaA multimer which is strictly necessary for initiation to occur [6]. But also other factors, one of them the Fis protein, have been suggested to be involved in this process.

The Fis protein was first known to be a factor for inversion stimulation in site-specific recombination and was later shown to be an important factor in transcriptional activation of genes involved in translation (see [7] for review) and a transcription factor involved in the regulation of a number of genes [8], [9]. The level of Fis has been shown to be high during exponential growth, but to drop to almost zero when the cells enter stationary phase [10]. The Fis protein has also been shown to bind within the origin of replication, *oriC*, in *E.coli* [11]–[15]. *In vitro* experiments have indicated an inhibitory role for the Fis protein in the control of replication [16], [17]. However, later it was shown that only high levels of Fis led to inhibition of replication *in vitro* and that this effect probably was due to altering of the supercoiling in the origin region [18]. Also, *in vivo* experiments have shown that deletion of the *fis* gene leads to perturbation of initiation timing in cells grown in LB medium [19] and to inhibition of DNA synthesis at elevated temperatures [12]. Hence, the *in vivo* results indicate a stimulatory role for this protein in the initiation of replication, at least under certain conditions.

Although it is known that a complex of the DnaA initiator protein has to be formed at the origin for initiation to occur and that other proteins, such as the Fis protein, affect this process, the details concerning the interplay of these different proteins in the initiation process is not yet fully understood. It is also not known whether the *E.coli* cells, which can have widely different growth rates and different replication patterns depending on the nutrients available [20], [21], regulate this process differently when grown under different conditions.

Here, we attempt to clarify the role of the Fis protein in the process of initiation of replication. The knowledge regarding the role of the Fis protein in this process comes mostly from *in vitro* replication assays [13], [14] and foot-printing assays [11], [15]. To shed more light on the role of the Fis protein in the initiation process *in vivo*, we have here used flow cytometry to investigate cells lacking the Fis protein or the Fis binding sites in the origin after growth in different media. We found that loss of the Fis protein did not have any effect in cells grown slowly in acetate medium. The Fis protein was however found to be important during more rapid growth, where it was shown to have a stimulatory role in the initiation process.

## Results

### The Fis protein is not required for initiation during slow growth in acetate, but is necessary for timely initiations during more rapid growth

**Figure 1:**
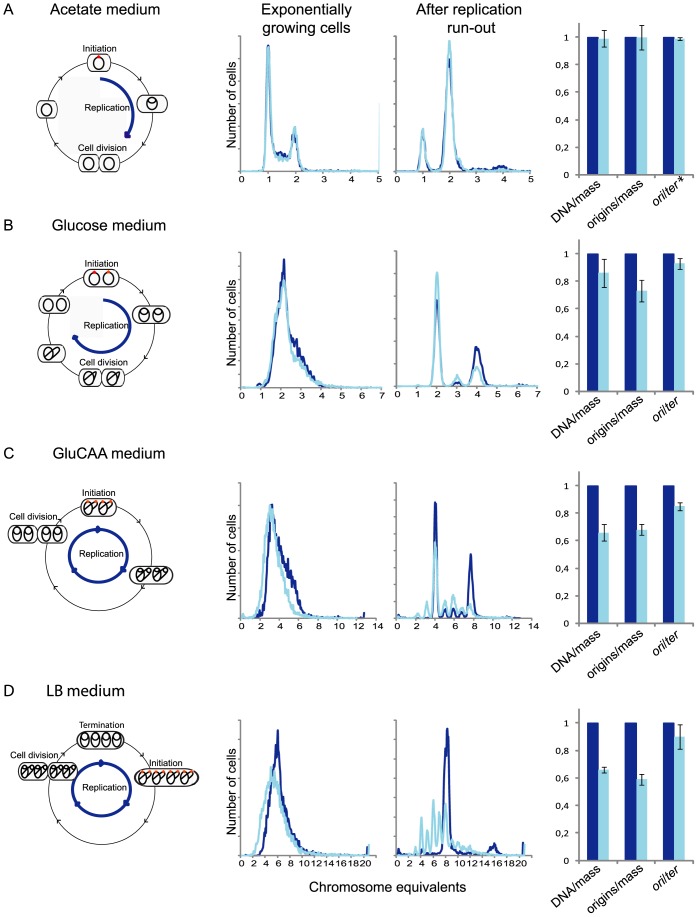
The Fis protein is necessary for timely initiations during rapid growth. Exponentially growing wild type and Δ*fis* cells and cells treated with rifampicin and cephalexin were analyzed by flow cytometry. The first (left-most) panels illustrate the replication patterns of wild type cells (see Materials and Methods for details of determination) grown at 30°C in (A) acetate medium, (B) glucose medium, (C) GluCAA medium and (D) LB medium. Cells with chromosomes (black lines) were drawn schematically to show the number of origins (red dots) initiated in the different media. In acetate grown cells initiation of replication occurred at one origin and the replication period (blue arrow) was completed within one generation (A). In the glucose, GluCAA and LB grown cells initiation of replication occurred at two, four and eight origins, respectively (B–D) and the replication spans more than one generation. The second and third panels show overlays of representative DNA histograms of exponentially and rifampicin/cephalexin treated wild type cells (dark blue) and Δ*fis* cells (light blue). Chromosome equivalents per cell are represented on the abscissa and the number of cells on the ordinate. 10000 cells were analyzed in each experiment. The values for DNA/mass, origin/mass and the origin to terminus ratio for the Δ*fis* cells relative to the wild type are shown in the bar histograms in panel four (A–D). The values are an average from three or more experiments and the error bars represent the standard deviation. *For the cells grown in acetate the origin to terminus ratio is determined from the DNA histogram of exponentially growing cells.

Wild type cells and cells where the *fis* gene had been exchanged with a kanamycin marker [22] were grown in four different media at 30°C to investigate the effect of the *fis* deletion on DNA replication at different growth rates. Samples of exponentially growing cells and cells treated with rifampicin and cephalexin were analyzed with flow cytometry (see Materials and Methods). In the poorest medium, minimal medium supplemented with acetate, the cells grow slowly with doubling times of around 4 hours (Table 1). Replication then initiates at one origin and is finished before cell division (Fig 1A, first panel). The cells therefore contain one, between one and two, and two chromosomes respectively through the cell cycle (Fig 1A, middle panels). In this medium the cells lacking the Fis protein grew with about the same doubling time as the wild type cells, had about the same DNA content (Fig 1A and Table 1) and no significant difference could be seen in the DNA histograms after flow cytometry (Fig 1A, middle panels). The experiment was repeated also at 37°C in the same medium and in media with glycerol as the carbon source. No differences between the wild type cells and the cells without the Fis protein were detected (data not shown). These results show that lack of Fis protein does not affect the timing of initiation of replication during slow growth.

**Table 1:** DNA, origin and DnaA concentrations of wild type and Δ*fis* cells grown in different media.

Strain	Carbon source	Doubling time	DNA/mass*	origin/mass*	DnaA conc.*
MG1655	Acetate	264 min	1	1	1
WM2652	Acetate	282 min	0.99	1.00	1.27
MG1655	Glucose	77 min	1	1	1
WM2652	Glucose	78 min	0.86	0.73	1.47
MG1655	GluCAA	51 min	1	1	1
WM2652	GluCAA	53 min	0.66	0.68	2.21
MG1655	LB	35 min	1	1	1
WM2652	LB	39 min	0.66	0.59	1.98

*relative to the wild type.

To see whether the lack of Fis protein affected more rapidly growing cells, the Δ*fis* cells were also grown in minimal medium supplemented with glucose (glucose medium), glucose and casamino acids (GluCAA medium) or LB medium at 30°C. In these media the cells have doubling times that are shorter than the time the cells need to replicate and segregate the chromosome so the replication of the chromosome starts in one of the previous generations. Cells grown in glucose medium initiate at two origins in the mother cell, cells grown in GluCAA at four origins in the grandmother cell and cells grown in LB medium at 8 origins in the great grandmother cell (Fig 1B–D, first panel). The cell cycle then spans 2, 3 or 4 generations respectively [21], [23].

In these media a change was observed in the cells lacking the Fis protein. The Δ*fis* cells grew with about the same doubling time as the wild type (Table 1) but exhibited decreased DNA and origin concentration (Fig 1B–D, fourth panel and Table 1). This shows that the Δ*fis* cells produce less DNA/mass than the wild type cells, i.e. they under-initiate. The histograms of the cells that have been treated with rifampicin and cephalexin (replication run-out) show that the number of fully replicated chromosomes is irregular and lower in the Δ*fis* cells compared to the wild type cells (Fig 1B–D, third panel). This phenotype became more pronounced with increasing growth rate. The most straightforward interpretation of the phenotype is that coordinated initiation at the right time becomes increasingly difficult with increasing growth rate. Previous work has shown that under-replication caused by under-initiation leads to an increase in average replication fork velocity [24]–[26]. In a culture with increased replication fork speed the ratio of origin to terminus markers will decrease. We determined the amount of the *oriC* and *ter* regions in all cell cultures (Fig 1, fourth panel). In an exponentially growing wild type culture the ratio of the *oriC* and *ter* region gives a direct measure of the duration of the replication period, C, from the formula *oriC*/*ter*  =  2^C/τ^, where τ is the doubling time (see Materials and Methods). For the cells grown in glucose, GluCAA and LB medium at 30°C the *oriC*/*ter* ratios were slightly lower for the Δ*fis* cells compared to the wild type cells (Fig 1, fourth panel). This indicates that the average C-period may be shorter in these cells compared to the wild type, which again could be a result of delayed initiation.

**Figure 2:**
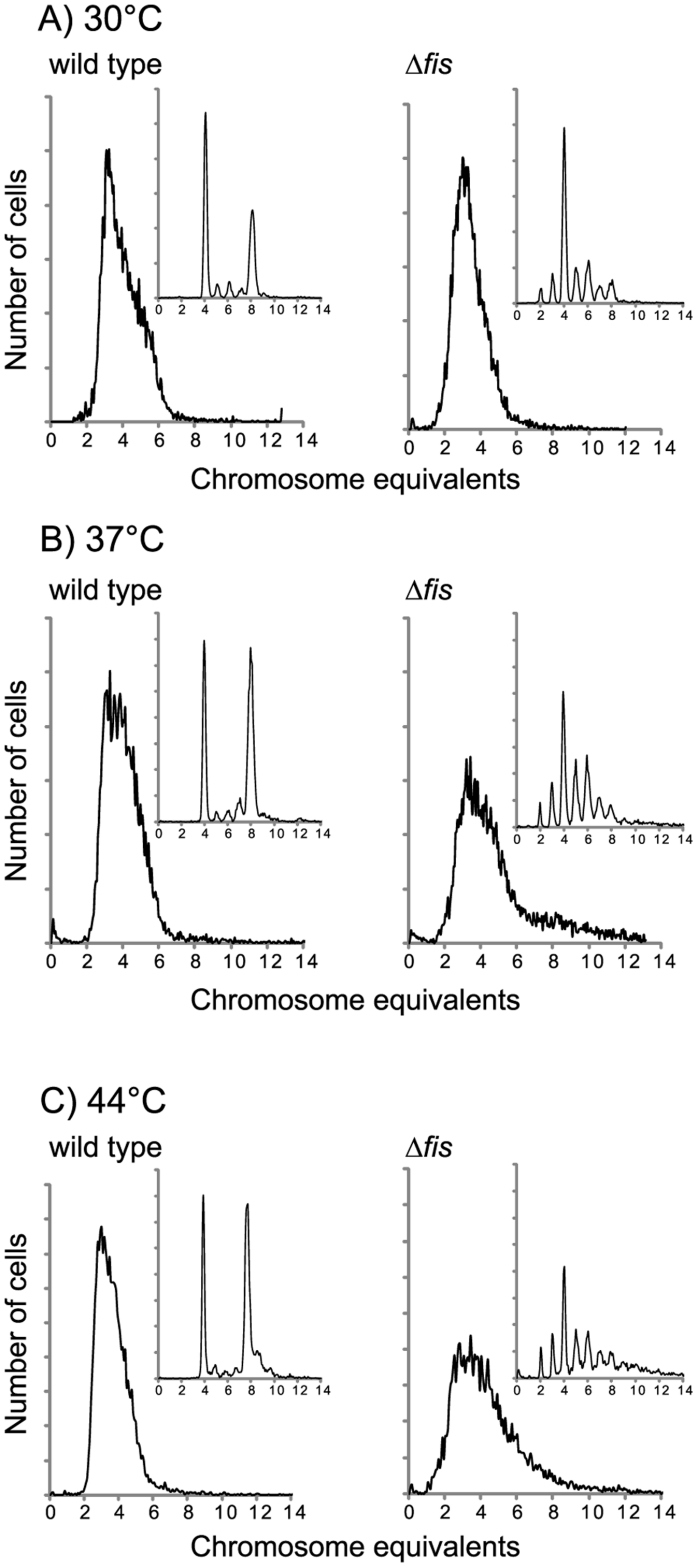
The effect of temperature on cells lacking the Fis protein. Flow cytometry DNA histograms of wild type and Δ*fis* cells grown in GluCAA medium at 30, 37 and 44°C. Chromosome equivalents per cell are represented on the abscissa and the number of cells on the ordinate. 10000 cells were analyzed in each experiment. Histograms of the replication run out samples (see Materials and Methods) are shown as small panels.

It has previously been reported that cells without the Fis protein form filaments and cease DNA replication at higher temperatures (44°) [12]. We therefore grew the cells in GluCAA medium also at 37°C and 44°C to see the effect of the temperature. We observed that the asynchrony was enhanced at the higher temperatures (Fig 2) and that the *oriC/ter* ratios were decreased with between 40 and 50% in the Δ*fis* cells compared to the wild type cells (data not shown). At the increased temperatures (but not at 30°C) we also observed filamentous cells, although not as extensive as previously reported [12] (data not shown). The lower *oriC*/*ter* ratios found at these temperatures might therefore be a result of non-replicating cells in the population. In either case the lower ratio indicates that the cells are having problems initiating replication; that they are either initiating much later than the wild type cells or that they cannot initiate at all. Also in LB we saw that an increase in the temperature led to increased asynchrony and lower *oriC*/*ter* ratio (data not shown).

The results presented here indicate that the Fis protein is necessary for properly timed and synchronous initiation of replication during rapid growth in glucose, GluCAA and LB medium, but is not required for the same processes during slow growth in acetate medium.

### The Δ*fis* cells under-initiate in spite of an increased level of the initiator DnaA

The Fis protein has been shown to regulate the expression of many genes and it is therefore likely that the Δ*fis* cells have altered levels of some proteins compared to the wild type. One of the genes regulated by the Fis protein is the *dnaA* gene [27]. The DnaA protein is one of the main actors in the initiation process and lack of DnaA can lead to under-initiation [28]. Therefore, the DnaA concentration in the wild type and Δ*fis* cells was measured. It was found to be elevated in the mutant at all growth conditions tested, from a 27% increase during growth in acetate medium, to 47% in glucose medium, and about 100% in GluCAA and LB medium (Table 1). This means that the cells lacking the Fis protein under-initiate during growth in glucose, GluCAA and LB medium in spite of an increased concentration of the initiator protein DnaA. This result further supports a possible stimulatory role for the Fis protein in the initiation of replication.

### The amount of Fis per cell is increased with increased growth rate

Previously it has been shown that the level of the Fis protein varies with the different growth phases, the level being high in exponential phase and low or non-existent in stationary phase [10], [29]. It was also shown that *fis* expression was up-regulated in cells after a nutritional up-shift [29]. We therefore checked if the cellular level of Fis varied during steady state growth in the media tested here and found that this was the case. In cells grown in glucose, GluCAA and LB the level was 1.9, 2.8 and 3.9 times higher than in the cells grown in acetate (Table 2). The cell mass also varies under different growth conditions. The mass was therefore measured by flow cytometry (as total protein content) and the Fis concentration expressed as the amount of Fis per cell. We then found that the amount of Fis per cell was 3.8, 6.7 and 14.3 higher in glucose, GluCAA and LB respectively, compared to cells grown in acetate (Table 2). Also the amount of Fis protein per origin was higher in the richer media (Table 2). This could indicate that the Fis protein is more important during rapid growth, in accordance with the above findings.

**Table 2:** Cellular concentration of Fis in wild type cells at different growth rates.

Carbon source	Fis conc.*	Mass*	Fis/cell	Average number of origins*	Fis/origin
Acetate	1	1	1	1.5 (1)	0.67
Glucose	1.90	2.0	3.8	2.7 (1.8)	1.41
GluCAA	2.81	2.4	6.7	5.9 (3.9)	1.14
LB	3.97	3.6	14.3	9.1 (6.1)	1.57

*relative to cell grown in acetate.

### Disruption of either Fis binding site I or II have different effects on regulation of initiation

**Figure 3:**
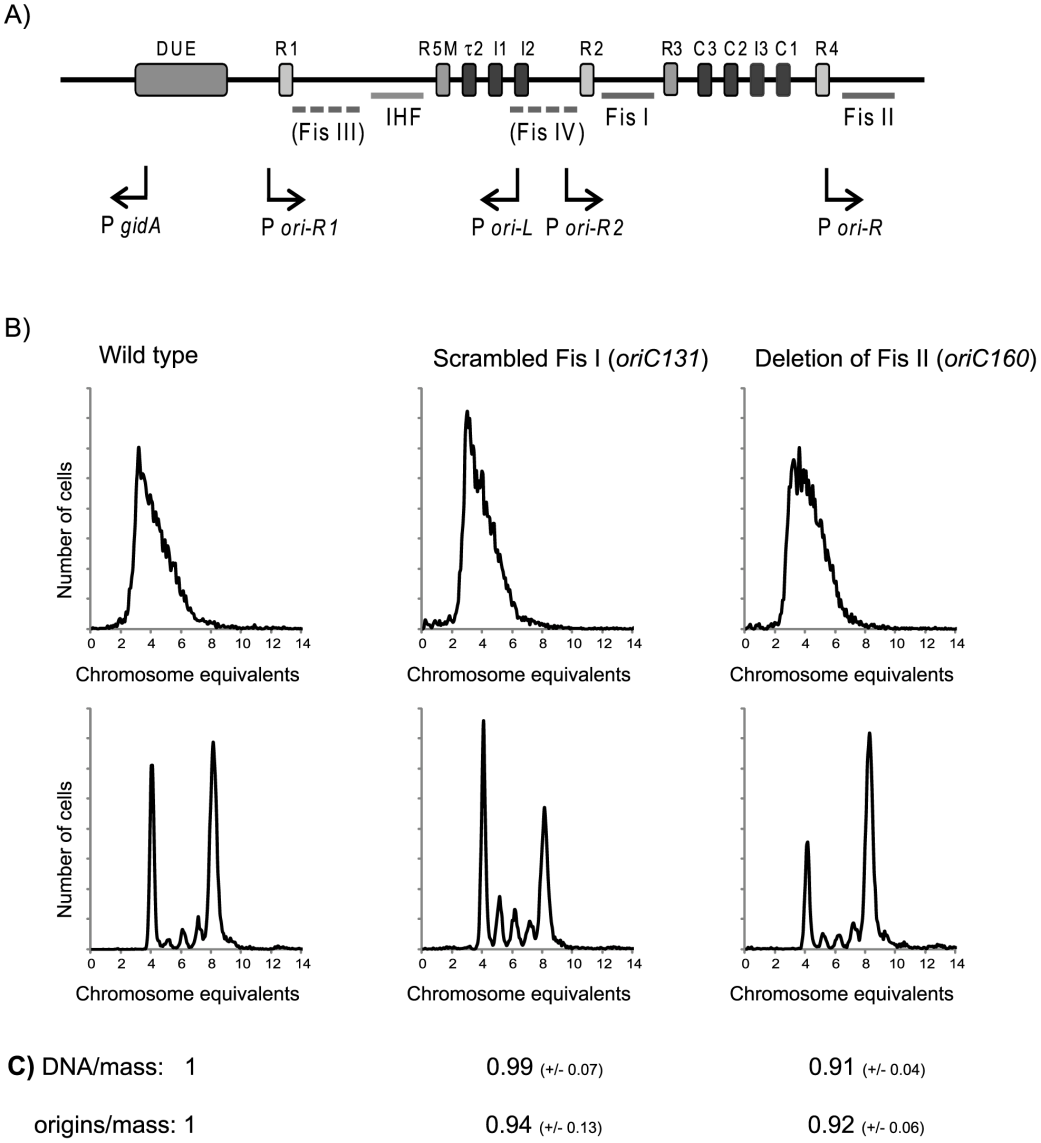
The effect of disruption of Fis binding site I or II on the regulation of initiation. A) An illustration of the origin region of *E.coli*. The high affinity DnaA boxes are shown in light grey and the lower affinity and ATP-DnaA boxes in dark grey. Binding sites and protected regions for the IHF and Fis protein are shown as horizontal lines. Promoters in the origin region are shown as arrows indicating the direction of transcription. B) DNA histograms of the exponential and replication run out samples of wild type cells and cells where the Fis site I had been scrambled (*oriC131*) or the Fis site II removed (*oriC160*) after growth in GluCAA medium at 37°C. The chromosome equivalents are represented on the abscissa and the number of cells on the ordinate. C) Relative values of DNA/mass and origin/mass for the two mutants relative to the wild type. The values are an average from three or more experiments and the standard deviations are given in parentheses.

Fis has been reported to bind to the origin and has two known binding sites (Fig 3A, Fis site I and II) but also other regions in the origin have been observed to be protected after binding of Fis (Fig 3A, Fis site III and IV) [12]. In order to investigate whether binding of Fis to the two known sites is important for the role of the Fis protein in the initiation process we investigated two different mutants where one of these Fis binding sites had been scrambled (*oriC*131) or removed (*oriC*160) [30]. These two strains were initially reported to have no phenotype when grown in GluCAA [30], but were later reported to have a higher number of origins compared to the wild type after growth in GluCAA medium [31]. The reason for this discrepancy is not known.

Here, we investigated these two mutants with flow cytometry after growth in acetate, glucose and GluCAA medium. For the cells grown in acetate and glucose medium no significant differences in the DNA histograms between the wild type cells and the cells lacking either of the two binding sites were observed (data not shown). However, after growth in GluCAA at 37°C we observed a slight change in the DNA histograms of the two mutants.

Scrambling of the Fis site I between DnaA box R2 and R3 (Fig 3A) led to cells that initiated at about the same time in the cell cycle as the wild type, but initiations were slightly asynchronous (Fig 3B, *oriC131*). This could indicate that this site might be important for simultaneous initiations at all origins present, but the effect is rather small compared to deletion of the *fis* gene.

The removal of the rightmost Fis site (Fig 3A) by deletion of 77 base pairs to the right of DnaA box R4 resulted in fewer cells with four origins compared to the wild type (Fig 3B, *oriC160*). This means that these cells initiate slightly earlier in the division cycle. These cells also had a 10% higher cell mass compared to the wild type giving a 10% reduction in the DNA and origin concentration (Fig 3C). Thus, these cells under-replicate. This result indicates that the Fis site II may be required for the stimulatory role of the Fis protein in the initiation process.

## Discussion

### The Fis protein is not important for regulation of initiation during slow growth, but essential for synchronous initiation at the right time during rapid growth

Several previous findings have indicated an inhibitory role for the Fis protein with regard to control of initiation [11], [16], [17]. Based on this and *in vitro* foot printing data showing that Fis bound to its primary site (Fis site I in Fig 3A) inhibits the binding of DnaA and IHF to *oriC* [15], Leonard and Grimwade proposed a model for the initiation complex assembly involving the Fis protein as an inhibitory element [32]. In this model the Fis protein is bound to the origin for the most of the cell cycle but is displaced by a build-up of DnaA oligomers at the R2 box, allowing binding of IHF. Binding of IHF presumably introduces a bend in the DNA that brings the weaker sites in closer proximity to the strong sites, causing a re-distribution of the ATP-DnaA molecules to the weaker sites subsequently followed by strand opening [32].

In the present work lack of Fis protein was shown not to influence initiation of replication in slowly growing cells, but to be important for proper timing of initiation of replication during rapid growth. If the Fis protein was a negative regulator of the initiation of replication as proposed in the model above, one would expect cells where the *fis* gene is deleted to over-initiate due to the lack of inhibition. However, the opposite result was found. When the Fis protein was lacking, the cells had a decreased DNA/mass and a lower number of origins compared to the wild type. The Δ*fis* cells were also unable to initiate the origins in synchrony. These results indicate that the role of the Fis protein is not entirely as presented in the model, but must also involve a stimulatory activity in the initiation process.

Removal of the Fis protein will probably lead to some changes in the protein composition in the cell, Fis being a transcription factor [8], [9]. Among the genes regulated by Fis is the gene encoding the DnaA protein, which was found to be up regulated in the absence of Fis. DnaA is one of the main actors in the initiation process and a build-up of DnaA molecules in the origin region is necessary for strand opening to occur [33]. Thus, an elevated level of DnaA would be thought to lead to earlier initiation. However, we found that, during rapid growth, the Δ*fis* cells under-initiated in spite of an elevated concentration of DnaA, a result that supports the idea of a Fis mediated stimulation in the initiation process.

**Figure 4:**
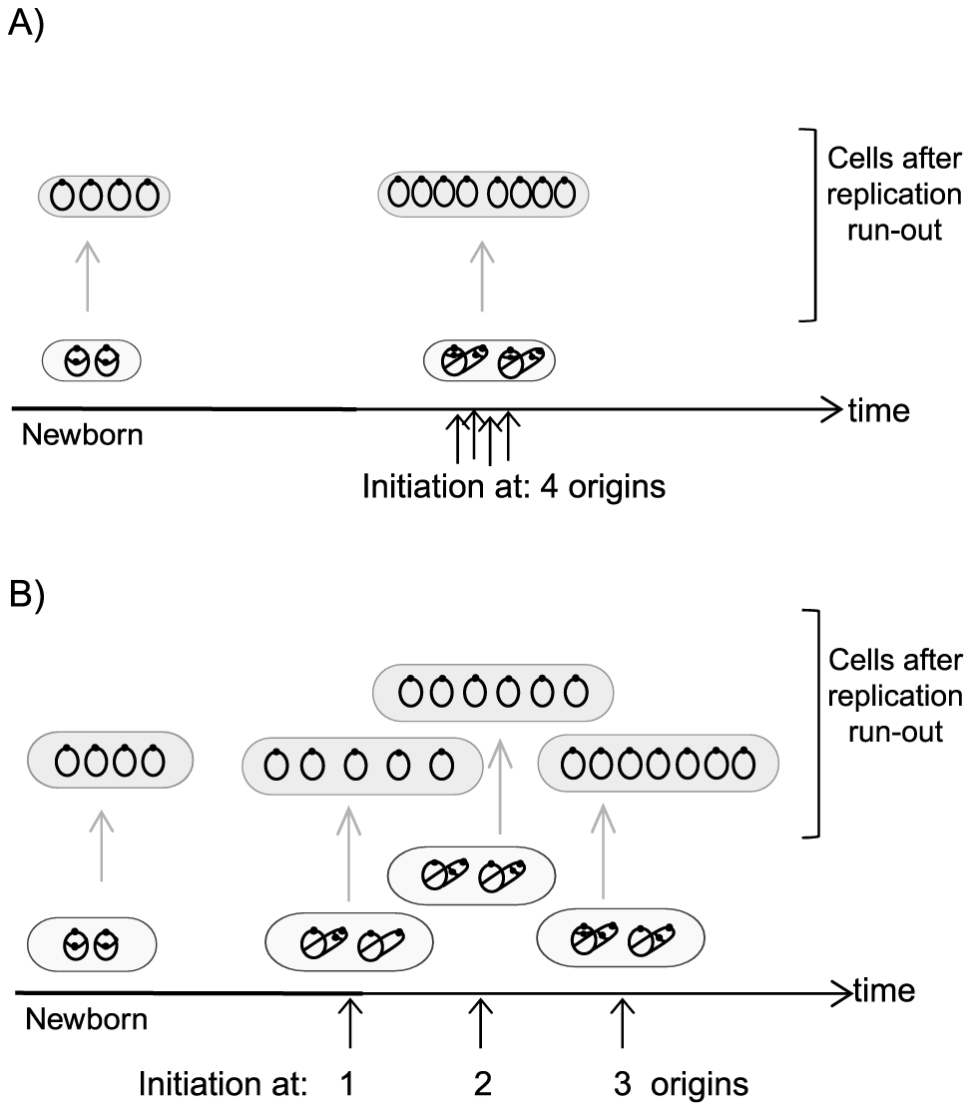
Schematic illustration of the replication pattern in wild type cells and in cells exhibiting under-initiation and asynchronous initiations. **A**) In a population of wild type cells, in which initiation occurs at four origins in synchrony, the cells will end up with either four fully replicated chromosomes (representing the cells that had not initiated at the time the drugs were added) or eight fully replicated chromosomes (representing the cells that had initiated at the time where the drugs were added) after treatment with rifampicin and cephalexin (grey cells). **B**) In a population with mutant cells that are exhibiting too few and untimely initiations the cells will end up with a number of fully replicated chromosomes that is not equal to 2^n^ or 2^n+1^, the actual number depending on how many of the origins had been initiated at the time the drugs were added. Here, an example where the newborn cell had 4 origins, but only three is initiated in the current generation, is shown.

Our results show that lack of Fis leads to a delay in the initiation at some of the origins in the cell and that some origins are not initiated at all. One example (of many) of such under-initiation is illustrated in Figure 4. In a wild type cell grown in GluCAA medium, as shown here, all four origins are initiated at the same time in the cell cycle. When such cells are treated with rifampicin and cephalexin they will end up with either four or eight fully replicated chromosomes after the drug treatment. The cells with four chromosomes are the cells that had not initiated at the time the drugs were added, while the cells that had already initiated at that time point will end up with eight whole chromosomes (Fig 4A and Fig 1C, third panel).

In contrast, when the synchrony of initiation is lost, like in the Δ*fis* cells, this will lead to a larger time interval for the initiation process (Fig 4B). In the example of the Δ*fis* cells, shown in Fig 4B, the newborn cell contains four origins like in the wild type situation. But in contrast to the wild type these are not all initiated at the same time, but one after the other when the cell has managed to form an initiation complex without the Fis mediated stimulation. Therefore, not all origins will have initiated at the time rifampicin and cephalexin is added, leading to cells with 4, 5, 6, 7 or 8 chromosomes in the run-out histogram (Fig 4B and Fig 1C, third panel). The average number of origins was also lower for the Δ*fis* cells, meaning that some of the origins are not initiated before the cell divides and would therefore be born with a lower number of origins (2 or 3). However, this must necessarily lead to extra initiations at some point because otherwise the number of origins per cell in the population would gradually decrease to zero and not be maintained at a steady average number. The net result in the Δ*fis* cells is that fewer origins are initiated per generation. In GluCAA medium wild type cells have on average 5.9 origins whereas the Δ*fis* cells have 5.1 origins per cell, and the difference is larger when the origin concentration is calculated (Fig 1C, fourth panel).

When the primary Fis site (Fis site I in Fig 3A) was scrambled the cells exhibited a slight asynchrony. This could suggest that binding of Fis to this site might be involved in the regulation of initiation. Deletion of Fis site II led to a slight shift in the initiation of replication to an earlier time point in the cell cycle and to a 10% reduction in DNA concentration. Thus, cells lacking this Fis site under-initiate. This result indicates that binding of Fis to *oriC* Fis site II has a direct stimulatory effect. The lack of large effect after the removal of either binding site I or II might be due to binding of Fis elsewhere in the origin, either at the other known binding site or at the other regions that have been shown to be bound by the Fis protein [12].

Our results are also supported by previous work suggesting a stimulatory role for the Fis protein in the initiation of replication. Bates and co-workers found that cells where the DnaA box R4 in the origin is deleted needed the Fis protein to be viable. They found that an *oriC* region lacking DnaA box R4 could not be transferred to a Δ*fis* strain unless it also contained the *rnhA*204 mutation which allowed the cell to initiate in an alternative manner from RNA:DNA hybrids [34]. Filutowicz *et al* found that Δ*fis* cells form filaments and that DNA replication ceases at high temperatures. In this work it was suggested that a Fis mediated stimulation must occur either before or at the transcriptional step in the initiation process [12]. This would be at a time point between binding of the DnaA molecules to the low affinity sites in the origin and the recruitment of DnaB [35]. One possible hypothesis is that the Fis protein, being a transcription factor, could stimulate the RNA polymerase in the transcriptional step needed for initiation to occur. There are several promoters within the origin (Fig 3A) and transcription from some of these could stimulate the unwinding process by altering the supercoiling [36]. With the exception of the *gidA* promoter, all these promoters are in close proximity of Fis binding sites (Fig 3A). A possible hypothesis might therefore be that Fis bound to one of these sites stimulates transcription occurring within the origin and hence the unwinding of *oriC*. If so, the Fis protein would first have an inhibitory role where it keeps the origin in the pre-replication complex state as proposed by Leonard and Grimwade [32], [37] and later a stimulatory role with regard to the transcription step required for strand opening.

### The control of initiation of replication differs with the growth rate/growth medium

We show here that whereas the Fis protein is dispensable during growth in acetate medium, cells lacking the Fis protein cannot initiate in an orderly manner in richer media. These results show that the factors needed in the regulation of the initiation of replication in the cells may vary during growth under different conditions. This is perhaps not a surprising finding as the growth medium and the growth rate affects the chemical composition of the cells [38] and also give rise to different replication patterns [21], [23]. During slow growth in acetate the cells initiate replication at one origin and complete the replication before division, whereas during more rapid growth the cells initiate at two or more origins in one of the previous generations leading to overlapping replication cycles. Such rapidly growing cells might need more extensive control mechanisms to ensure that each origin is initiated only once per cell cycle [1], [39], [40]. Another difference between cells grown in different media is the amount of initiator available. We have previously shown that cells growing slowly in acetate medium have more DnaA available per origin than cells grown in GluCAA [41]. Thus, these cells might need less stimulatory factors (like Fis) in order to build a functional initiation complex compared to cells with less DnaA available per origin.

The need for transcription in the initiation of replication might also vary between different growth conditions. So another possible explanation for the fact that the slowly growing cells do not react to lack of the Fis protein might be that these cells are not so dependent on the transcription in the origin region in order to initiate. Lack of stimulation of transcription would then not be a problem for such cells. In *Escherichia coli* rifampicin, a drug that inhibits transcription can be used to inhibit new initiations. Slowly growing cells, in contrast to cells grown in rich media, have been found to exhibit rifampicin resistant rounds of replication [41], a fact that supports this idea.

## Materials and Methods

### Bacterial strains, plasmids and growth conditions

All strains used are *Escherichia coli* K-12 and are listed in Table 3. Cells were grown in LB medium or AB minimal medium [42] supplemented with 10 µg/ml thiamine, 25 or 100 µg/ml uridine and either 0.4% sodium acetate (acetate medium), 0.2% glucose (glucose medium) or 0.2% glucose and 0.5% casamino acids (GluCAA medium) as the carbon source at 30°C, 37°C or 44°C. 25 µg/ml kanamycin was added when necessary. The growth rates were determined by measuring the optical density of the cultures at 600 or 450 nm. Cultures to be diluted in acetate medium were first grown to OD∼0.3 in minimal medium supplemented with 0.1% glucose and then diluted to OD∼0.0025 in acetate medium.

**Table 3:** Strains and plasmids used in this study.

Strain	Relevant genotype	Reference
MG1655	Wild type	[45], [46]
WM2652	MG1655 *fis::km*	[22] 1
ALO2256	MG1655, wild type	[31],[30]
ALO2257	MG1655 *oriC160*	[31],[30]
ALO2262	MG1655 *oriC131*	[31],[30]

*fis::km* from the CSH50 background in [22] to MG1655 in the Messer research group.^1^ WM2652 was made by P1 transduction of the

### Quantitative PCR of the *oriC* and *ter* region

To obtain the ratios of the *oriC* and *ter* region, chromosomal DNA was purified from exponential cultures (OD∼0.15). Quantitative PCR performed as described in [43] and normalized to a sample where the *oriC*/*ter* ratio was 1∶1. The experiment was repeated three or more times and the standard deviation calculated.

### Flow cytometry analysis and calculation of cell cycle parameters

Exponentially growing cells (OD∼0.15) were harvested and fixed with ethanol or treated with 300 µg/ml rifampicin and 10 µg/ml cephalexin for two or more generations before fixation. The DNA was stained with Hoechst 33258 (Sigma-Aldrich). The total protein in the cells was stained with Fluorescein isothiocyanate (FITC, Sigma-Aldrich) and used to calculate the average mass. Flow cytometry was performed with a LSR II flow cytometer (BD Biosciences).

Treatment with rifampicin and cephalexin inhibits new rounds of initiation and cell division, respectively, but allows ongoing replication to finish [40]. The DNA histograms of such cells (so called run out histograms) will contain peaks of cells with a number of fully replicated chromosomes corresponding to 2^n^ and 2^n+1^ where n is a number between 0 and 3 and depends on the generation where replication is initiated. n = 0 if initiation occurs in the current generation, n = 1 if it occurs in the mother generation and n = 2 or 3 if initiation occurs in the grandmother or great grandmother generation respectively. The number of fully replicated chromosomes reflects the number of origins present in the cell at the time the drugs were added [39], [40].

When all cells in a population are in exponential growth (with the same generation time and the same replication pattern), the cell cycle parameters (initiation age and replication period) can be calculated by combining the data from the flow cytometry analysis, the theoretical age distribution of an exponential culture and the generation time obtained by OD measurements. This was done in an excel based simulation program [44]. The length of the replication period can also be calculated from the ratio of *oriC* to *ter* using the formula *oriC*/*ter*  =  2^C/τ^, where τ is the doubling time obtained by OD measurements. All experiments were repeated three times or more.

### Quantification of Fis and DnaA concentrations

Exponentially growing cells (OD∼0.15) were harvested and treated with SDS. Samples were subjected to SDS-polyacrylamide gel electrophoresis and western blot was performed using rabbit anti-DnaA-antibody or anti-Fis-antibody (laboratory stocks) and ECF fluorescence kit (GE Healthcare). Quantification was performed using Image Quant software (Molecular Dynamics).
